# The controlled direct effect of temperament at 2-3 years on cognitive and academic outcomes at 6-7 years

**DOI:** 10.1371/journal.pone.0204189

**Published:** 2019-06-04

**Authors:** Shiau Yun Chong, Catherine Ruth Chittleborough, Tess Gregory, John Lynch, Murthy Mittinty, Lisa Gaye Smithers

**Affiliations:** 1 School of Public Health, University of Adelaide, Adelaide, Australia; 2 Robinson Research Institute, Adelaide, Australia; 3 Telethon Kids Institute, University of Western Australia, Perth, Australia; 4 Population Health Sciences, University of Bristol, Bristol, England, United Kingdom; Temple University, UNITED STATES

## Abstract

There is widespread interest in temperament and its impact upon cognitive and academic outcomes. Parents adjust their parenting according to their child’s temperament, however, few studies have accounted for parenting while estimating the association between temperament and academic outcomes. We examined the associations between temperament (2–3 years) and cognitive and academic outcomes (6–7 years) when mediation by parenting practices (4–5 years) was held constant, by estimating the controlled direct effect. Participants were from the Longitudinal Study of Australian Children (n = 5107). Cognitive abilities were measured by the Peabody Picture Vocabulary Test (verbal) and the Matrix Reasoning test (non-verbal). Literacy and numeracy were reported by teachers using the Academic Rating Scale. Mothers reported children’s temperament using the Short Temperament Scale for Toddlers (subscales: reactivity, approach, and persistence). Parenting practices included items about engagement in activities with children. Marginal structural models with inverse probability of treatment weights were used to estimate the controlled direct effect of temperament, when setting parenting to the mean. All temperament subscales were associated with cognitive abilities, with persistence showing the largest associations with verbal (PPVT; β = 0.58; 95%CI 0.27, 0.89) and non-verbal (Matrix Reasoning: β = 0.19; 0.02, 0.34) abilities. Higher persistence was associated with better literacy (β = 0.08; 0.03, 0.13) and numeracy (β = 0.08; 0.03, 0.13), and higher reactivity with lower literacy (β = -0.08; -0.11, -0.05) and numeracy (β = -0.07; -0.10, -0.04). There was little evidence that temperamental approach influenced literacy or numeracy. Overall, temperament had small associations with cognitive and academic outcomes after accounting for parenting and confounders.

## Introduction

There is widespread interest in whether children’s temperament influences their cognitive and academic outcomes [1, 2]. Temperament is one of many concepts captured under the phrase ‘non-cognitive’ skills, which are believed to improve human capital through improved cognitive, academic, social and health outcomes. Temperament is the individual characteristics in behavioral styles that are biologically-based, but also shaped by experiences and environment [3]. Three aspects of temperament thought to impact learning and cognition are reactivity, persistence and approach [4]. Reactivity encompasses a child’s emotional intensity and volatility [4]. For instance, a child who regularly shows emotional irritability is considered as having high emotional reactivity. Persistence reflects the ability to stay ‘on task’[5] despite distractions or difficulties. Temperamental approach is the degree of comfort experienced when encountering new situations or people [4]. For example, a child who withdrew or was wary to a novel environment is considered as having low temperamental approach. These three aspects of temperament have potential to influence children’s cognitive abilities and academic achievement. In our recent systematic review and meta-analysis of non-cognitive abilities, we reported that there were very few high quality studies relating various measures of temperament to either cognition or academic achievement [6].

With respect to cognition, the overall concept of temperament has been associated with cognitive outcomes in some [7, 8] but not all [9] studies. This suggests that certain components of temperament may have specificity for cognitive outcomes. Components of temperament such as higher persistence have been linked to better cognitive outcomes among American children [10]. There is also some indication that effects are consistent across cultural settings, where measures of persistence have been linked to better development among Japanese children [11].

For academic achievement, aspects of temperament such as high emotional reactivity may interfere with the child’s learning processes [1, 12]. For example, a highly reactive child may become easily frustrated and find it difficult to learn [13]. Whereas temperamental traits such as persistence are more likely to help a child stay on task and maintain their attention despite distractions [4], which has benefits for learning [14]. Low approach may present challenges for young children in the transition to school as they are faced with many new situations, teachers and peers [15].

Reactivity and persistence are two aspects of temperament that are linked to a child’s self-regulation skills. Self-regulation refers to the ability to regulate emotions, manage behaviors, focus attention in the face of distraction and to be able to persist at a task [13]. This definition highlights the interconnectedness of temperament, self-regulation, executive functioning (i.e. the cognitive control of one’s behaviors in order to achieve a goal) and attentional control. It is through these links that temperamental reactivity and persistence are likely to affect cognitive ability and academic achievement [16]. Previous research has shown that self-regulation skills in general and emotional reactivity and persistence specifically have been linked to cognition and academic achievement [13, 17, 18]. For instance, a review by Blair & Raver [17] showed that dimensions of self-regulation including emotional regulation and executive function are important skills needed for school readiness and early academic achievement. West, Denton, Germino-Hausken [18] found that teacher-rated children’s task persistence is related to reading, mathematics, and general knowledge in 22,000 American kindergarten students. While there may be a direct effect of temperament on child cognitive and academic outcomes, children’s temperament may also influence the types of parenting they received. For instance, a study of 35 pairs of mother-child showed that mothers displayed more negative and non-accepting behaviors to children with low attention span [19]. In addition, parents may also engage in fewer playing or reading activities with a child who shows emotional distress, and this could in turn influences the child’s cognitive and academic outcomes [20]. Hence the importance of considering parenting when evaluating whether aspects of temperament are associated with children’s outcomes.

Temperamental reactivity and persistence are modifiable [21, 22]. For instance, a cluster-randomized trial showed that an intervention to develop children’s persistence, attention, and impulse control resulted in improvements to academic outcomes [22]. Increasing children’s persistence and reducing their reactivity may be a mechanism to improve children’s cognitive and academic outcomes, provided that there are direct effects of temperament on these outcomes [21–23].

To estimate the direct effect of temperament, we need to account for the fact that children’s temperament may influence parenting [24, 25], which in turn, is known to influence children’s cognitive and academic outcomes [26]. Maccoby *et al* [25] showed that temperamentally difficult children (*i*.*e*. high emotional intensity, difficult to calm) received less teaching from parents at 18 months than temperamentally easy children. However, Dixon *et al* [24] found mothers engaged in more high quality play with temperamentally difficult than easy children. Parental engagement in play and cognitive stimulation activities has positive impacts on children’s outcomes [26], and differential parental engagement for temperamentally easy and difficult children might, in part, explain effects of temperament on cognitive and academic outcomes.

While some studies have examined the direct effect of temperament on cognitive and academic outcomes, most involve limited adjustment for confounding [2, 12, 27]. The few studies that accounted for parenting suggest an association between temperament and outcomes [20, 28, 29]. However, simple adjustment for parenting practices could introduce bias when parenting practices are affected by temperament (parenting is a mediator) and when there are confounders of parenting and outcomes (mediator-outcome confounding; S1 File) [30]. In the current study, we use traditional linear regression models to examine the total effect of temperament (reactivity, approach, persistence) at 2–3 years on cognitive and academic outcomes at 6–7 years, simply to compare results with past studies. In our main findings, we use marginal structural models (MSMs) to estimate what are called “controlled direct effects” (CDEs) [30] of temperament on cognitive and academic outcomes, while accounting for parenting practices at 4–5 years. Although these estimates are called controlled direct effects in the epidemiological literature, where interpretation is required, these estimates are referred to as associations. As the focus of our research is the association between temperament and cognitive and academic outcomes, rather than the mediated effect through parenting, the CDE is the most appropriate estimate to use. MSMs adjust for confounding using inverse probability of treatment weighting [31]. MSMs allow control of parenting by setting it to some uniform value, which in turn, enables the estimation of the ‘controlled’ direct effect of temperament on outcomes. Specifically, parenting is not the variable of interest, we need to account for it but it is not part of the effect we want to estimate. Another advantage of MSMs over traditional methods is weighting for mediator-outcome confounding, as this does not involve statistical adjustment for the intermediate variable, which could introduce bias (S1 File) [32].

## Methods

### Study design and sample

Data were from the Longitudinal Study of Australian Children (LSAC). LSAC is a population-based study that commenced in 2004. Participants were recruited using a two-stage clustered sampling process [33]. At commencement, 5107 infants (mean age 8.8 months) were recruited and followed-up at 2–3 (n = 4606), 4–5 (n = 4386), and 6–7 (n = 4242) years. LSAC is considered broadly representative of Australian children [33].

### Ethical approval and consent

All procedures performed in studies involving human participants were in accordance with the ethical standards of the institutional and/or national research committee and with the 1964 Helsinki declaration and its later amendments or comparable ethical standards. LSAC was approved by the Australian Institute of Family Studies ethics committee. Written informed consent was obtained from all participants’ caregivers.

### Data

The authors do not have the authority to provide data to researchers. However, de-identified data used in the current study from LSAC is accessible to *bona fide* researchers by application. Further information on how to access the data is available at the following webpage (https://growingupinaustralia.gov.au/data-and-documentation/accessing-lsac-data).

### Cognitive ability and academic achievement (*Y*)

Verbal ability (receptive vocabulary) was measured using an adapted Peabody Picture Vocabulary Test (PPVT). The adapted PPVT-III [34] was administered by a trained interviewer to children aged 6–7 years during home interviews. The child pointed to the picture that best represented the meaning of a word spoken by the examiner [35]. The adapted PPVT-III was comparable to the full PPVT-III (correlations ranging 0.93–0.97) with high internal consistency (person-separation reliability 0.76) [34]. Scale scores were created using Rasch modelling (Mean = 64, standard deviation (SD) = 8) [34].

Non-verbal ability (fluid reasoning) was measured using the Matrix Reasoning test from the Wechsler Intelligence Scale for Children, 4^th^ edition [36]. The Matrix Reasoning test comprised 35 items. The child was presented with an incomplete set of diagrams and asked to select the picture that completes the set from 5 different options. Scores were reported as standard scores, from age-appropriate norms (mean = 10, SD = 3). High internal consistency of the Matrix Reasoning test has been established in normative samples of Australian children (Cronbach’s α = 0.88 for 6-year-olds, 0.91 for 7-year-olds) [36].

Academic achievement at 6–7 years was measured using the adapted Academic Rating Scale (ARS) [37], which has two subscales: literacy (10 items) and numeracy (9 items). Teachers rated the child’s skills and knowledge in relation to other children of the same age from ‘not yet demonstrated skill’ to ‘demonstrates skill competently and consistently’. Examples of literacy items included ‘reads books fluently’ and ‘writes sentences with more than one clause’. Numeracy items included ‘uses a variety of strategies to solve math problems’ and ‘makes reasonable estimates of quantities’. Total scores ranging from 1 to 5 were created using Rasch modelling. Higher scores indicate higher proficiency. The ARS has high internal reliability (Cronbach’s α = 0.96 for literacy, 0.95 for numeracy) [38].

### Temperament (*X*)

Temperament was measured at 2–3 years using the Short Temperament Scale for Toddlers (STST) [39]. The STST was adapted from the Toddler Temperament Scale [40]. The STST consists of 3 subscales (4 items each): reactivity, approach, and persistence, rated by the primary caregiver (98.2% mothers) from 1 (almost never) to 6 (almost always). Average scores were calculated for each subscale. Higher scores indicate higher reactivity (more negative emotion), higher approach (lower shyness), and higher persistence. All subscales had acceptable internal reliability in the current sample (α = 0.76 for approach, 0.68 for reactivity and 0.75 for persistence).

### Parenting (*M*)

Parenting was assessed at 4–5 years using the home activities index which contains 7 items measuring how often mothers read to the child, tell stories, draw pictures, play indoor games, outdoor games, music, and involve the child in activities such as cooking or pet care. These items have been used as indicators of the quality of home environment, such as frequency of parent-child activities in UNICEF surveys [41]. Items were rated from 0 (none) to 3 (everyday), and a total score (0–21) derived by summing item scores (internal reliability α = 0.71). Higher scores indicates more positive parenting practices.

### Confounders of the association between temperament and outcomes (*C*)

Factors that might confound the associations between temperament and cognitive and academic outcomes were decided *a priori* using a directed acyclic graph (S2 File). Confounders included indicators of socioeconomic position, intrauterine, child, maternal and family factors. These confounders were reported by mothers when children were 0–1 year. Details about how these confounders were measured are included in S2 File.

### Confounders of the association between parenting practices and outcomes (*L*)

To estimate the CDEs, we need to account for confounding associated with parenting and cognitive or academic outcomes [30]. This set of confounders were reported by mothers at ages 4–5 years and included variables that were affected by temperament and in turn confound the parenting-outcome association (maternal psychological distress, number of siblings, maternal working status, household income, and financial hardship).

### Analysis

MSMs are recommended for overcoming the limitations of standard regression models. They allow estimation of the direct effect in the presence of mediators and confounders of mediator-outcome associations, and allow interaction between the exposure and mediator[30]. The MSM differs from standard regression models because the MSM is a model for potential outcomes rather than observed outcomes [30, 42]. A potential outcome is the outcome that an individual would have had under some different level of the exposure. If the observed and potential outcomes differ, it is assumed that all other things being equal, the exposure has caused the difference in outcome. However, it is usually only possible to observe one outcome for an individual and therefore the potential outcome is not observed for that same individual. The potential outcome is also referred to as the counterfactual outcome when it is different from the observed value. This terminology is widely used in the field of causal inference, within the discipline of epidemiology.

The ‘marginal’ in the name of the *marginal* structural model refers to the fact that the estimated effects are marginal, not conditional as in standard regression. Theoretically, the marginal effect is the difference in outcome (*Y*) when the exposure *X* is set to a level x and a counterfactual level x* for each individual. In this analysis we used weighted regression to estimate the average of the differences in the potential outcomes to derive the direct effect. In the current study we are interested in the direct effect of temperament and not the mediated effect. A key reason for using the MSM in this instance is that it correctly accounts for mediation (by parenting) leading to less biased estimates of the association between temperament and cognitive and academic outcomes, than would be generated using standard regression analyses (S1 File).

We tested three models. Model 1 estimated the total effect of temperamental reactivity, approach or persistence (*X*) on cognitive and academic outcomes (*Y*) after adjusting for confounders (*C*) using standard linear regression. Model 2 included further adjustment for mediators (*M*) and confounders (*C*, *L*) in standard linear regression. Most studies have used standard regression models 1 and 2, and these are provided for comparison with previous work. However, our *a priori* primary analysis (model 3) involved MSM. Using MSMs, we estimated the CDEs of temperament (reactivity, approach, or persistence, *X*), on cognitive and academic outcomes (*Y*) after accounting for parenting practices *(M)*, potential confounders of the association between temperament and cognitive and academic outcomes *(C)*, and confounders of parenting practices and outcomes (*L*). As described above the standard regression estimates the conditional effect, whereas the MSM weighted regression estimates the marginal effect of temperament on cognitive and academic outcomes. The marginal effect is the difference in cognitive and academic outcomes under the observed (*x*) and counterfactual exposures (x*) of each individual. The mediator *M* (parenting) is set to a uniform level of *m*, such that the associations between temperament and outcomes are not mediated by parenting.[30] We used the log-likelihood ratio to test for interactions between temperament and parenting. As no interactions were found, the CDE generates the same result regardless of the level at which the mediator is set. Therefore, we set the mediator to its mean value (*m*). The CDEs were estimated from linear regression models of the form:

$$E\left[Y_{xm}\right]=E[Y_{i}\text{|}X_{i}=x_{i},M_{i}=m\text{]}=\beta_{0}+\beta_{1}x_{i}+\beta_{2}m$$ (1)

Potential confounding was accounted by fitting the model above with stabilized inverse probability weights of the form $=w_{i}^{X}\times w_{i}^{M}$, where

$$w_{i}^{X}=\frac{f\left(X_{i}\right)}{f\text{(}X_{i}\text{|}C_{i})}$$ (2)

and

$$w_{i}^{M}=\frac{f\text{(}M_{i}\text{|}X_{i})}{f(M_{i}|X_{i},C_{i},L_{i})}.$$ (3)

The weight $w_{i}^{X}$ accounted for the confounding of the association between temperament and cognitive and academic outcomes by conditioning on *C*. The weight for the mediator $w_{i}^{M}$ accounted for confounding of the association between parenting and cognitive and academic outcomes by conditioning on *X*, *C*, and *L*. Since the mediator was a continuous variable, probabilities were taken from the density functions. For computing the probabilities we assume that the mediator is normally distributed and hence used the normal density function. In the normal density function, parameters used to obtain probabilities were the observed mediator value, the predicted means and the root mean square error, estimated from linear regression [30]. Weights were truncated at the 1^st^ and 99^th^ percentile to deal with outliers (S1 File). MSMs were performed separately for temperament reactivity, approach, and persistence. Effect estimates were reported as unstandardized β coefficients and in SD units, which are calculated by dividing the unstandardized β by the SD of the imputed sample.

Associations from standard regressions and MSMs were estimated under the assumption that there was no unmeasured confounding between the outcome and exposure, or between the mediator and the outcome. However, as these assumptions are unverifiable we performed sensitivity analysis to determine the extent to which an unmeasured confounder *U* might affect the association between temperament and cognitive and academic outcomes. We estimated the bias for the CDEs under conditions varying in prevalence and effect size of *U* (S3 File) [43].

We tested sex by exposure interactions. As no significant interactions were found, analyses were conducted on the full sample.

Analyses were performed using STATA 13.0 (StataCorp, College Station, TX).

### Multiple imputation

A non-monotonic pattern of missingness was observed. We generated twenty imputed datasets under the missing at random assumption [44]. The imputation model included temperament, parenting practices, cognitive and academic outcomes, confounding variables and auxiliary variables that predicted missingness (parenting self-efficacy, temperament sociability, persistence, and reactivity at 4–5 years). We also performed analyses on the sample with observed outcome and the results were similar to the imputed sample. Results from the imputed sample (n = 5107) are reported.

## Results

Table 1 shows the characteristics of LSAC response, complete case (*n* = 1647) and imputed (*n* = 5107) samples. The highest proportion of missing data was teacher-reported outcomes of literacy and numeracy, although data from over 3300 children were available for these outcomes. Characteristics of the imputed sample were similar to the response sample.

**Table 1:** Characteristics of response, complete case, and imputed samples^a^.

	Response sample^b^		Complete case sample^c^, n = 1647	Imputed sample, n = 5107
	N	M (SD) or %	M (SD) or %	M (SD) or %
**Outcomes, *Y* (Cognitive ability and academic achievement)**				
PPVT	4185	74.4 (5.2)	75.2 (4.9)	74.2 (5.2)
Matrix reasoning	4180	10.7 (3.0)	11.0 (3.0)	10.7 (3.0)
ARS-Literacy	3408	3.4 (0.8)	3.5 (0.7)	3.4 (0.8)
ARS-Numeracy	3357	3.3 (0.8)	3.5 (0.8)	3.3 (0.8)
**Exposures, *X* (Temperament)**				
Reactivity	3530	3.0 (1.0)	2.9 (0.9)	3.0 (0.9)
Approach	3533	3.9 (1.0)	4.0 (1.0)	3.9 (1.0)
Persistence	3532	4.3 (0.7)	4.3 (0.7)	4.3 (0.7)
**Intermediate variable, *M***				
Parenting practices	4385	11.8 (3.9)	12.0 (3.8)	11.7 (3.9)
**Confounders of *X*-*M* or *X*-*Y***				
Mother’s highest education, %				
Tertiary	1677	32.9	41.4	32.9
Diploma/certificate	1766	34.6	34.2	34.6
Schooling only	1656	32.5	24.4	32.5
Hardship score	5089	0.9 (1.3)	0.7 (1.1)	0.9 (1.3)
Housing tenure, rented or other, %	5100	35.8	23.0	35.8
Aboriginal or Torres Straits Islander, %	5107	4.5	2.1	4.5
Index of relative socio-economic disadvantage (IRSD)	5107	1008.8 (60.2)	1017.2 (57.2)	1008.8 (60.2)
Child is male, %	5107	51.1	48.5	51.1
Birthweight for gestational age z-score	4999	0.0 (1.1)	0.1 (1.0)	0.0 (1.1)
Duration of breast feeding, %				
Never breastfed	420	9.2	5.8	9.2
<1 month	518	11.4	8.4	11.4
<3 months	473	10.4	8.7	10.3
<6 months	692	15.2	14.0	15.2
6 months or more	2461	53.9	63.2	54.0
Maternal age	5106	31.0 (5.5)	32.2 (4.6)	31.0 (5.5)
Mother is born in Australia, %	4997	80.0	83.0	79.4
Mother’s psychological distress	4308	4.4 (0.8)	4.5 (0.5)	4.4 (0.6)
Mother and partner argumentative relationship	3931	2.2 (0.6)	2.1 (0.6)	2.2 (0.6)
Single-parent household, %	5103	9.7	0.2	9.7
Mother had gestational hypertension, %	4238	7.8	7.8	8.1
Mother had gestational diabetes, %	4223	5.7	5.2	5.9
Any alcohol during pregnancy, %	4227	38.9	44.1	37.3
Smoking during pregnancy, %	4239	16.7	10.6	18.2
**Confounders of *M*-*Y***				
Mother’s psychological distress	3818	4.5 (0.6)	4.5 (0.5)	4.5 (0.6)
Number of siblings	4386	1.5 (1.3)	1.5 (0.9)	1.5 (1.0)
Mother’s working status, %				
Working full-time	957	21.8	19.8	21.1
Working part-time	1804	41.2	46.6	40.7
Currently not working	1622	37.0	33.6	38.2
Household income per week, $	3668	1977.8 (1343.4)	2054.5 (1365.3)	1893.6 (1297.7)
Hardship score	4365	0.3 (0.7)	0.2 (0.6)	0.3 (0.8)

^a^ Abbreviations: N, sample number; M(SD), mean (standard deviation); *Y*, outcomes; PPVT, Peabody Picture Vocabulary Test; ARS, Academic Rating Scale; *X*, exposures; *M*, mediators; *X-M*, exposure-mediator; *X-Y*, exposure-outcome; IRSD, index of relative socioeconomic disadvantage; *M-Y*, mediator-outcome

^b^ Response sample is the number of participants who responded to specific assessment for each child exposure, outcome, or confounder,

^c^Complete case sample are participants who have data for every variable (i.e. no missing on any variable.

Table 2 displays associations between temperament subscales and child outcomes using regression models adjusted for *C* (Model 1), *C*, *M*, and *L* (Model 2) and the MSM (Model 3). The CDEs estimated from the MSM (Model 3) were closer to total effects (Model 1), while estimates from conventional regression (Model 2) were lower. The MSM showed higher reactivity had negative associations with all outcomes, particularly verbal ability (PPVT; β = -0.37 95% CI -0.59, -0.14). Higher approach had positive associations with verbal and non-verbal abilities but little or no association with literacy or numeracy. Higher persistence had positive associations with all outcomes. Among the four outcomes, the largest associations were for verbal ability. In the MSM for instance, 1-unit higher persistence (range 1–5) was associated with 0.58-unit (0.11 SD) higher verbal (PPVT) and 0.19-unit (0.06 SD) higher non-verbal ability (Matrix Reasoning).

**Table 2:** Effect estimates of temperament reactivity, approach, and persistence at ages 2 to 3 years on child outcomes at ages 6 to 7 years (n = 5107)^a^.

	Model 1^b^, total effect			Model 2^b^, conventional regression			Model 3^b^, controlled direct effect		
	β^c^	(95% CI)	SD units	β^c^	(95% CI)	SD units	β^c^	(95% CI)	SD units
**Reactivity**									
PPVT	-0.38	(-0.59, -0.17)	-0.07	-0.36	(-0.56, -0.15)	-0.07	-0.37	(-0.59, -0.14)	-0.07
Matrix reasoning	-0.09	(-0.18, 0.01)	-0.03	-0.09	(-0.19, 0.002)	-0.03	-0.11	(-0.21, -0.01)	-0.04
ARS-Literacy	-0.07	(-0.10, -0.04)	-0.09	-0.07	(-0.10, -0.04)	-0.09	-0.08	(-0.11, -0.05)	-0.10
ARS-Numeracy	-0.06	(-0.09, -0.03)	-0.08	-0.06	(-0.09, -0.03)	-0.08	-0.07	(-0.10, -0.04)	-0.09
**Approach**									
PPVT	0.46	(0.28, 0.65)	0.09	0.40	(0.21, 0.59)	0.08	0.45	(0.22, 0.67)	0.09
Matrix reasoning	0.10	(0.01, 0.19)	0.03	0.08	(-0.01, 0.18)	0.03	0.11	(0.01, 0.21)	0.04
ARS-Literacy	0.03	(-0.01, 0.06)	0.04	0.02	(-0.01, 0.06)	0.03	0.03	(-0.01, 0.07)	0.04
ARS-Numeracy	0.02	(-0.02, 0.05)	0.03	0.01	(-0.03, 0.05)	0.01	0.01	(-0.02, 0.05)	0.01
**Persistence**									
PPVT	0.61	(0.38, 0.84)	0.12	0.55	(0.32, 0.79)	0.11	0.58	(0.27, 0.89)	0.11
Matrix reasoning	0.16	(0.04, 0.29)	0.05	0.17	(0.05, 0.30)	0.06	0.19	(0.02, 0.34)	0.06
ARS-Literacy	0.08	(0.04, 0.12)	0.10	0.08	(0.04, 0.12)	0.10	0.08	(0.03, 0.13)	0.10
ARS-Numeracy	0.08	(0.04, 0.12)	0.10	0.09	(0.05, 0.13)	0.11	0.08	(0.03, 0.13)	0.10

^a^ Abbreviations: CI, confidence interval; SD, standard deviation units; PPVT, Peabody Picture Vocabulary Test; ARS, Academic Rating Scale

^b^ Model 1 is adjusted for confounders, *C*. Model 2 is the conventional regression model including *X*, *M*, and all confounders *C* and *L*. Model 3 is a marginal structural model including *M* and weighted for all confounders *C* and *L*.

^c^ β are unstandardized coefficients

The sensitivity analysis showed that the CDEs were generally robust in the presence of a binary unmeasured confounder (S3 File). The observed CDEs would be explained by an unmeasured confounder if its prevalence differed between the exposed (*x*) and counterfactual (x*) by ≥80% and the estimated mean of the outcome differed by ≥0.60 within the two levels of the unmeasured confounder.

## Discussion

We found evidence that suggests an association between temperament at 2–3 years on children’s cognitive abilities and academic outcomes at ages 6–7 years. Of the three temperament dimensions, persistence had the largest association, where a 1-unit increase in persistence (5-point Likert scale) was associated with an increase of 0.11 SD for verbal ability, 0.10 SD for literacy and numeracy and 0.06 SD for non-verbal reasoning. These results are similar to studies that measured persistence using different questionnaires [1, 14], children of different ages and using different statistical approaches [1]. For instance, a cross-sectional study of effortful control measured using the Child Behavior Questionnaire (CBQ) at 3–5 years was associated with 0.29 SD higher letter knowledge and 0.17 SD math achievement [14]. Rudasill *et al* [27] reported attention at 4.5 years measured with the CBQ was associated with 0.18 SD higher reading scores and 0.14 SD mathematic scores in 8–10 year-olds.

Reactivity was negatively associated with cognitive and academic outcomes, with the largest association being 0.10 SD on literacy. This was similar to studies of preschoolers [1, 12] where CBQ temperament scores at 5.6 years from parents and teachers were averaged and standardized associations of ~0.18–0.28 SD on mathematics and reading were observed [12]. The similarity in findings reported in different studies from different countries, at different ages, as well as the use of different tools for measuring temperament adds strength to these findings.

Consistent with previous work involving the STST [15], higher scores on approach (lower shyness), were associated with higher verbal (0.09 SD) and non-verbal (0.04 SD) cognitive abilities. While we found little evidence of temperament approach on literacy and numeracy, others have reported that shy children were more likely to have poorer academic achievement [45]. Differences in these findings may be due to the small (n = 125) cross-sectional design or because shyness was measured at 9–13 years, when children were expected to have more developed sociability skills.

Few studies have accounted for parenting when investigating associations between temperament and cognitive and academic outcomes, where often only narrow aspects of parenting have been examined (e.g. involvement in schooling [28], joint attention [29]). We defined parenting as the frequency parents engaged in activities with their children because there is evidence this is important for children’s development [26]. This operationalization of parenting is limited because it only measures the frequency and not the quality of the parent-child interactions. The parenting measure does not include other actions by parents (such as harsh parenting, hitting) that might affect children’s development [46]. Furthermore, parenting activities were reported by parents themselves, which raises concerns about potential biases such as same-rater bias or social desirability bias. Same-rater bias may be attenuated by the 2-year period between parents reporting of temperament (age 2–3) and parenting (age 4–5). Although having the same rater might conflate the temperament-parenting *(X-M*) association, no interaction between temperament and parenting was detected. Possible social desirability bias might have been mitigated through the parent questionnaires being returned by post. Although we expect mothers to have excellent knowledge of their child’s temperament, it has been suggested that reporting of temperament might differ among mothers from lower socioeconomic position or suffering depression [47]. Broader definitions of parenting, temperament or use of different mediators might reveal different findings. Thus, although we accounted for parenting, there may be other pathways that temperament could influence cognitive and academic outcomes, such as peer and teacher relationships or child care. Given that the associations between temperament and cognitive and academic outcomes were small, future research could examine temperament within the context of parenting practices, for instance, the effect of parenting on cognitive and academic outcomes may be heightened in temperamentally difficult compared with easy children. Parenting interventions could specifically target children with difficult temperament if they were more susceptible to the impact of parenting on their cognitive and academic outcomes.

Strengths of this study include the use of a large nationally-representative sample, prospective follow-up, direct measures of cognitive outcomes and teacher-reports of academic outcomes. Previous studies have often been limited to small, non-representative samples, cross-sectional or short-term design (1–2 years) [6]. For instance, Valiente et al studied emotionality and academic abilities 6-months later in 291 children [12]. Furthermore, our methods account for parenting which is thought to influence children’s cognitive and academic outcomes, and adjust for a wide range of potential confounders. It has been proposed that teachers might grade children with more challenging temperamental traits less favorably [48–51]. Teacher-reported outcomes also reflect how children are perceived in “real world” settings and one reason why broader studies are needed on the potential impacts of temperament. Another strength of these findings is the consistency in the direction of associations for outcomes measured using direct assessments (verbal and non-verbal cognition) and outcomes reported by teachers (literacy and numeracy).

The temperament tool used here (the STST) was developed in an Australian sample and it was useable in a large population-based study. There are many different conceptualizations of temperament and its’ components, and it is possible that different temperament tools, or direct/laboratory based measures may result in different findings. Nevertheless, the results reported here are consistent with studies using different temperament tools.

To understand the practical implications of this work, we reported effect sizes in SD units (Table 2) to help compare the current study with other evidence. While we acknowledge the problems interpreting standardized effect sizes [52], the current study involves a nationally-representative sample. This means that the SDs on which the associations are standardized are likely to be relevant to population-based interventions, and are less likely to be exaggerated than studies from homogenous samples. So how do the effects reported here compare with interventions that aim to improve aspects of children’s regulation of temperament traits? Large, population-based interventions in school settings, such as PATHS and the Chicago School Readiness Program, report effect sizes in the order of 0.03–0.5SD for literacy outcomes [6, 22, 53, 54]. This is largely in line with what is reported here and with other observational studies [6]. Unfortunately, there are few rigorously tested interventions that can be scaled-up to the population-level that might improve academic outcomes, and this represents an area of future development.

In conclusion, the findings described in the present study suggest that temperamental traits such as persistence, approach and reactivity at 2–3 years of age may be linked to cognitive and academic outcomes at ages 6–7 years. As an indication of the magnitude of these associations, the largest association suggested that a 1-point increase in persistence was linked to a 0.11SD increase in verbal ability.

## Supporting information

S1 File The marginal structural model. (PDF)

S2 File Confounders of the association between temperament and outcomes. (PDF)

S3 File Sensitivity analysis for controlled direct effects. (PDF)
