# Supplementary material for: The controlled direct effect of temperament at 2-3 years on cognitive and academic outcomes at 6-7 years
Source: PLoS One. 2019 Jun 4;14(6):e0204189. doi: 10.1371/journal.pone.0204189 (PMC6548354; doi:10.1371/journal.pone.0204189)
Supplement: S1 File — (PDF) [file pone.0204189.s001.pdf]

Supporting Information. S1 File.

The controlled direct effect of temperament at 2-3 years on cognitive and academic outcomes at 6-7 years

Shiau Yun Chong[1], Catherine Ruth Chittleborough[1,2], Tess Gregory[1,3], John Lynch[1,2,4], Murthy Mittinty[1,2], Lisa Gaye Smithers[1,2]*

[1] School of Public Health, University of Adelaide, Adelaide, Australia

[2] Robinson Research Institute, Adelaide, Australia

[3] Telethon Kids Institute, University of Western Australia, Perth, Australia

[4] Population Health Sciences, University of Bristol, Bristol, England, United Kingdom

*Corresponding author

Email: lisa.smithers@adelaide.edu.au (LGS)

S1 File. The marginal structural model

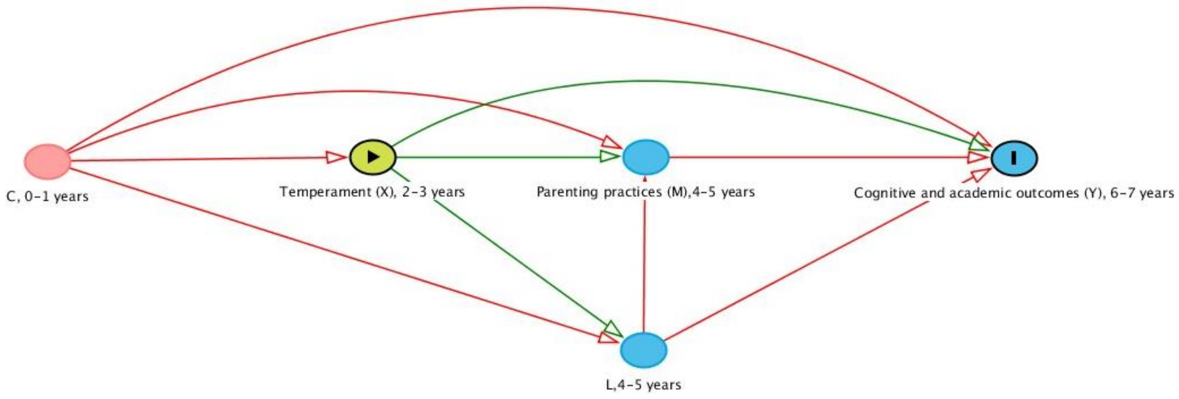

S1 Fig. Causal diagram of the hypothesized effects of temperament at 2 to 3 years and parenting practices at 4 to 5 years on cognitive and academic outcomes at ages 6 to 7 years.

S1 Fig depicts the causal diagram for our study. *X* (temperament subscales of reactivity, approach, and persistence) represents the exposure, *M* (parenting practices) represents the intermediate variable, and *Y* (cognitive and academic outcomes) represents the outcome. *C* represents confounders of the association between temperament (*X*), parenting practices (*M*), and cognitive and academic outcomes (*Y*) measured at ages 0 to 1 year (maternal education, financial hardship, housing tenure, Aboriginal or Torres Strait Islander, neighbourhood disadvantage, sex, birth weight for gestational age z-score, duration of breastfeeding, maternal age, maternal country of birth, maternal psychological distress, mother and partner argumentative relationship, single-parent family, gestational hypertension, gestational diabetes, smoking and alcohol intake during pregnancy). *L* represents confounders of the effect of parenting practices *M* on cognitive and academic outcomes *Y* measured at ages 4 to 5 years (maternal psychological distress, number of siblings, mothers' working status, household income, and financial hardship).

The standard approach to estimate the direct effect is by regressing the outcome *Y* on the exposure *X* and some exposure-outcome confounders *C* and then considering whether the coefficient for *X* changes when controlling for the intermediate variable *M*. The difference in coefficients of *X* is a measure of the effect that is going through by *M* [1]. However, using the standard regression approach to assess the direct effect can lead to biased estimates when there are confounders of the *M*-*Y* association, *L* [2, 3]. For example, the number of siblings *L* affects parenting practices *M* at age 4 to 5 years and child outcomes *Y* at age 6 to 7 years. If we adjusted for *M*, as in the regression approach, we induce an association between *X* and *L* [2]. If we additionally adjusted for *L*, we block part of the direct effect of *X* on *Y* that is not through *M* [4].

To overcome limitations of standard regression, marginal structural models have been recommended for better estimation of the controlled direct effect [5]. The marginal structural model differs from the standard regression approach in that the model is for counterfactual outcomes rather than observed outcomes [5, 6]. The counterfactual approach allows the estimation of the controlled direct effect by comparing the extent to which an outcome would change if the mediating variable (parenting practices) was controlled by setting at a uniform level *m* in the population while the exposure was changed from the observed level, *x* to a counterfactual level, x^* [5].

The marginal structural model is not conditioned on any covariates but uses a weighting approach to account for confounding factors [7, 8]. Under the assumption of no unmeasured confounding factors, the weighting method creates a pseudo population in which the association between confounding factors and the exposure is ignorable. The weighting

approach takes into account the confounding effect of L to allow a better estimation of the direct effect [9]. The creation of weights was based on four assumptions [10]:

1. Consistency - the potential outcome for every individual depends on his/her exposure history. For example, that the effect of temperament on PPVT is the same (i.e. consistent) for any level of temperament.

2. Conditional exchangeability - the outcome Y is independent of the exposure X, given the covariates. This assumption is also known as ‘no unmeasured confounding’. We examined this assumption in the sensitivity analysis.

3. Positivity - both exposed and unexposed individuals are present at every level of the confounders.

4. Correct model misspecification - the model used to create the weights was appropriate (*e.g.* linear relationship, interaction term included if appropriate, and sufficient confounding factors). It is possible that there is some model misspecification, for example, if the distribution of the exposure/mediator residuals was skewed, the weights generated for the exposure and mediator from normally distributed probability density function may be biased. **Table S1** displays the estimated stabilized inverse probability weights.

Table S1: Stabilized inverse probability weights

	**Estimated weight**		**Weight truncated at 99th percentile**	
	**Mean (SD)**	**Minimum/maximum**	**Mean (SD)**	**Minimum/maximum**
Reactivity	1.00 (0.55)	0.03/54.12	0.99 (0.38)	0.25/4.39
Approach	1.00 (0.46)	0.04/41.36	0.99 (0.32)	0.27/3.54
Persistence	1.00 (0.47)	0.05/33.10	0.99 (0.31)	0.29/3.30

References for S1 File

1. Baron R, Kenny D. The moderator-mediator variable distinction in social psychological research: Conceptual, strategic, and statistical considerations. J Pers Soc Psychol. 1986;51(6):1173-1182.
2. Cole S, Hernan MA. Fallibility in estimating direct effects. International Journal of Epidemiology. 2002;31:163-165.
3. Robins JM, Greenland S. Identifiability and exchangeability for direct and indirect effects. Epidemiology. 1992;3(2):143-155. doi: 10.2307/3702894.
4. Daniel R, De Stavola B, Cousens S. gformula: Estimating causal effects in the presence of time-varying confounding or mediation using g-computation formula. The Stata journal. 2011:1-30.
5. VanderWeele TJ. Marginal structural models for the estimation of direct and indirect effects. Epidemiology. 2009;20:18-26.
6. Hernan MA. A definition of causal effect for epidemiological research. Journal of Epidemiology and Community Health. 2004;58:265-271.
7. Cole SR, Hernán MA. Constructing inverse probability weights for marginal structural models. American Journal of Epidemiology. 2008;168(6):656-664. doi: 10.1093/aje/kwn164.
8. Naimi A, Moodie EE, Auger N, Kaufman J. Constructing inverse probability weights for continous exposures: A comparison of methods. Epidemiology 2014;25(2):292-299.
9. Robins JM, Hernan MA, Brumback B. Marginal structural models and causal inference in epidemiology. Epidemiology. 2000;11(5):550-560.
10. Cole S, MA H. Constructing inverse probability weights for marginal structural models. American Journal of Epidemiology. 2008;168:656-664.11.
