# Supplementary material for: The controlled direct effect of temperament at 2-3 years on cognitive and academic outcomes at 6-7 years
Source: PLoS One. 2019 Jun 4;14(6):e0204189. doi: 10.1371/journal.pone.0204189 (PMC6548354; doi:10.1371/journal.pone.0204189)
Supplement: S2 File — (PDF) [file pone.0204189.s002.pdf]

# Supporting Information. S2 File.

The controlled direct effect of temperament at 2-3 years on cognitive and academic outcomes at 6-7 years

Shiau Yun Chong[1], Catherine Ruth Chittleborough[1,2], Tess Gregory[1,3], John Lynch[1,2,4], Murthy Mittinty[1,2], Lisa Gaye Smithers[1,2]*

[1] School of Public Health, University of Adelaide, Adelaide, Australia

[2] Robinson Research Institute, Adelaide, Australia

[3] Telethon Kids Institute, University of Western Australia, Perth, Australia

[4] Population Health Sciences, University of Bristol, Bristol, England, United Kingdom

*Corresponding author

Email: lisa.smithers@adelaide.edu.au (LGS)

# S2 File. Confounders of the association between temperament and outcomes

Indicators of socioeconomic position included maternal education (tertiary, diploma and certificate, and schooling only), housing tenure (owned/mortgaged versus rented/other), Aboriginal or Torres Strait Islander (yes/no), financial hardship, and neighborhood disadvantage. Financial hardship was assessed from 7 items asking whether mothers had experienced the following due to shortage of money: adults or children went without meals; they were unable to heat or cool their home; they pawned or sold something; or they sought assistance from a welfare or community organization. Hardship scores ranged from 0 to 7, with a higher score indicating greater financial difficulties. The Index of Relative Socio-economic Disadvantage (IRSD) was used as an indicator of neighborhood disadvantage. The IRSD is based on postcode of residence and has a national mean of 1000 and a standard deviation of 100, with lower scores indicating greater disadvantage [1].

Intrauterine factors included self-reported gestational hypertension (yes/no), gestational diabetes (yes/no), and smoking and alcohol intake during pregnancy (yes/no). Child factors included sex, birthweight for gestational age z-score and duration of breast feeding (never breastfed, < 1 month, <3 months, < 6 months, and ≥6 months). Birthweight percentiles were calculated based on Australian birthweight percentiles data collected from 1998 to 2007 stratified by sex and gestational age [2].

Maternal and family factors included age, maternal country of birth (Australia versus other countries), psychological distress, mother and partner argumentative relationship, and single-parent household (yes/no). Mother's psychological distress was assessed using the Kessler

K6 scale with a total score that ranged from 1 to 5 [3]. After recoding, higher scores indicated less distress. Five questions were asked about conflicts the mother had with her partner, including disagreement, argument, stressful conversation, verbal hostility, and physical hostility. Responses for each item ranged from 1 (not at all) to 5 (all the time). Higher scores indicated more argumentative relationships.

# References for S2 File

1. Australian Bureau of Statistics. Socio-economic indexes for areas (SEIFA) 2011. Australia: Commonwealth of Australia; 2013.
2. Dobbins T, Sullivan E, Roberts C, Simpson J. Australian national birthweight percentiles by sex and gestational age, 1998–2007. Med J Aust. 2012;197(5):291-294.
3. Kessler RC, Green JG, Gruber MJ, Sampson NA, Bromet E, Cuitan M, et al. Screening for serious mental illness in the general population with the K6 screening scale: Results from the WHO World Mental Health (WMH) survey initiative. Int J Meth Psychiatr Res. 2010;19:4-22. doi: 10.1002/mpr.310.
