# Supplementary material for: The controlled direct effect of temperament at 2-3 years on cognitive and academic outcomes at 6-7 years
Source: PLoS One. 2019 Jun 4;14(6):e0204189. doi: 10.1371/journal.pone.0204189 (PMC6548354; doi:10.1371/journal.pone.0204189)
Supplement: S3 File — (PDF) [file pone.0204189.s003.pdf]

Supporting Information. S3 File.

The controlled direct effect of temperament at 2-3 years on cognitive and academic outcomes at 6-7 years

Shiau Yun Chong[1], Catherine Ruth Chittleborough[1,2], Tess Gregory[1,3], John Lynch[1,2,4], Murthy Mittinty[1,2], Lisa Gaye Smithers[1,2]*

[1] School of Public Health, University of Adelaide, Adelaide, Australia

[2] Robinson Research Institute, Adelaide, Australia

[3] Telethon Kids Institute, University of Western Australia, Perth, Australia

[4] Population Health Sciences, University of Bristol, Bristol, England, United Kingdom

*Corresponding author

Email: lisa.smithers@adelaide.edu.au (LGS)

S3 File. Sensitivity analysis for controlled direct effects

Sensitivity analyses were conducted to assess the effect of an unmeasured confounder, *U* of the association between *X* and *Y*, on the CDEs of the temperament subscale of reactivity on cognitive and academic outcomes. Assuming that *U* is a binary variable and the effect of *U* on *Y* on the additive scale, conditional on exposure, mediator, and covariates (*X*, *M*, *C*) is the same for both exposure levels X=*x* and X=*x**, the bias for the conditional CDE is defined as [1]:

$$Bias\ \left(CDE_{x,x*}(\mathrm{m})\right) = \delta\gamma \qquad (1)$$

Where δ denotes the difference in prevalence of *U* in the exposed, *x* and counterfactual, *x** group while γ denotes the effect size of *U* on cognitive and academic outcomes.

Table S2 showed that to invalidate the observed CDE (β=-0.37) of reactivity on the PPVT score, the unmeasured confounder *U* would need to have a difference in prevalence of 80% (for example 90% for exposure level *x*, and 10% for counterfactual exposure level *x**) and would be required to decrease the PPVT score by at least 0.6. *U* might have an effect size of 0.6 on the PPVT score. However, it does not appear plausible for a *U* to have a prevalence difference of 0.8 or more that could eliminate the CDE of reactivity on PPVT.

Table S2. The effect of a potential unmeasured binary confounder *U* of parenting practices to outcomes pathway

P(U=1|x, m, c)[(1)]	*P(U=1|x*, m, c*[(2)]	*δ=*[(1)-(2)]	*γ*	*d=δγ*
**Peabody Picture Vocabulary Test (direct effect of reactivity β=-0.37)**				
0.6	0.4	0.2	-0.40	-0.08
0.7	0.3	0.4	-0.40	-0.16
0.8	0.2	0.6	-0.40	-0.24
0.9	0.1	0.8	-0.40	-0.32
0.6	0.4	0.2	-0.60	-0.12
0.7	0.3	0.4	-0.60	-0.24
0.8	0.2	0.6	-0.60	-0.36
0.9	0.1	0.8	-0.60	-0.48
0.6	0.4	0.2	-0.80	-0.16
0.7	0.3	0.4	-0.80	-0.32
0.8	0.2	0.6	-0.80	-0.48
0.9	0.1	0.8	-0.80	-0.64
**Matrix Reasoning Test (direct effect of reactivity β =-0.11)**				
0.6	0.4	0.2	-0.10	-0.02
0.7	0.3	0.4	-0.10	-0.04
0.8	0.2	0.6	-0.10	-0.06
0.9	0.1	0.8	-0.10	-0.08
0.6	0.4	0.2	-0.20	-0.04
0.7	0.3	0.4	-0.20	-0.08
0.8	0.2	0.6	-0.20	-0.12
0.9	0.1	0.8	-0.20	-0.16
0.6	0.4	0.2	-0.30	-0.06
0.7	0.3	0.4	-0.30	-0.12
0.8	0.2	0.6	-0.30	-0.18
0.9	0.1	0.8	-0.30	-0.24
**ARS-Literacy (direct effect of reactivity β =-0.08)**				
0.6	0.4	0.2	-0.05	-0.01
0.7	0.3	0.4	-0.05	-0.02
0.8	0.2	0.6	-0.05	-0.03

0.9	0.1	0.8	-0.05	-0.04
0.6	0.4	0.2	-0.10	-0.02
0.7	0.3	0.4	-0.10	-0.04
0.8	0.2	0.6	-0.10	-0.06
0.9	0.1	0.8	-0.10	-0.08
0.6	0.4	0.2	-0.15	-0.03
0.7	0.3	0.4	-0.15	-0.06
0.8	0.2	0.6	-0.15	-0.09
0.9	0.1	0.8	-0.15	-0.12
**ARS-Numeracy (direct effect of reactivity β =-0.07)**				
0.6	0.4	0.2	-0.05	-0.01
0.7	0.3	0.4	-0.05	-0.02
0.8	0.2	0.6	-0.05	-0.03
0.9	0.1	0.8	-0.05	-0.04
0.6	0.4	0.2	-0.10	-0.02
0.7	0.3	0.4	-0.10	-0.04
0.8	0.2	0.6	-0.10	-0.06
0.9	0.1	0.8	-0.10	-0.08

$P(U = 1|x, m, c)$ = prevalence of the unmeasured confounder for exposure level *x*; $P(U = 1|x^*, m, c)$ = prevalence of the unmeasured confounder for exposure level *x**. *δ* = difference in the prevalence of the unmeasured confounder between exposure level *x* and *x**; *γ*= beta coefficient of the direct effect of U on *Y*; d =magnitude of bias, is the product of *δ* and *γ*.

Reference for S3 File

1. VanderWeele TJ. Bias formula for sensitivity analysis for direct and indirect effects. Epidemiology 2010;21(4):540-551.
